# A *PDLIM7* Variant in Familial Mitral Valve Prolapse: A Case Series

**DOI:** 10.1002/ccr3.70282

**Published:** 2025-03-28

**Authors:** Aniek L. van Wijngaarden, Tamara T. Koopmann, Claudia A. L. Ruivenkamp, Hoi W. Wu, Nina Ajmone Marsan, Daniela Q. C. M. Barge‐Schaapveld

**Affiliations:** ^1^ Department of Cardiology Leiden University Medical Center Leiden the Netherlands; ^2^ Department of Clinical Genetics Leiden University Medical Center Leiden the Netherlands

**Keywords:** cardiology, genetics and genomics, mitral valve prolapse, PDLIM7, whole exome analysis

## Abstract

In the presented case of familial mitral valve prolapse, whole exome sequencing was used to reveal a missense variant in the *PDLIM7* gene. This gene is considered a possible novel candidate gene for familial MVP based on PDLIM7 knock‐out mice and zebrafish showing mitral valve abnormalities.

## Graphical abstract

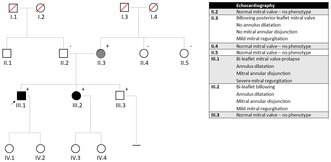

## 1 Introduction

Degenerative mitral valve disease causing mitral valve prolapse (MVP) is one of the most common valvular abnormalities, affecting 2%–3% of the population [1]. MVP can lead to severe mitral regurgitation requiring surgery or ventricular arrhythmias and even sudden cardiac death [2]. Multiple studies have suggested an important hereditary component of MVP [3, 4]. So far, few non‐syndromic causative genes have been identified with different types of inheritance patterns (*FLNA* [5] with recessive X‐linked inheritance pattern, *DCHS1* [6] and *DZIP1* [7] with autosomal dominant inheritance pattern and *PLD1* [8] with autosomal recessive inheritance pattern). Furthermore, several genes related to specific syndromes, such as Marfan syndrome (*FBN1* gene) [9] and trichorhinophalangeal syndrome (*TRPS1* gene) [10], have also been related to MVP. In a recent study based on large‐scale exome‐based analysis in more than 100 MVP patients, additional novel candidate genes, namely those associated with cardiomyopathy, were identified [11]. Despite these preliminary findings, most genetic causes of familial MVP are unknown, and the identification of novel candidate genes, such as the *PDLIM7* gene in the current report, will provide a better understanding of the MVP pathophysiology and will prove to be useful in family screening.

The PDLIM7 protein is a member of the PDZ‐LIM family, a family of proteins that are thought to act as signal mediators involved in a variety of cellular processes such as migration, signal transduction, and differentiation [12, 13]. PDLIM7 contains one PDZ and three LIM domains, is associated with cytoskeletal actin [14] and regulates the localization and activity of the nuclear transcription factor TBX5 [15]. PDLIM7 plays a role in cardiac development, and PDLIM7 knockdown in zebrafish and mice has been shown to result in valvular abnormalities [15, 16].

## 2 Case Examination

**FIGURE 1:**
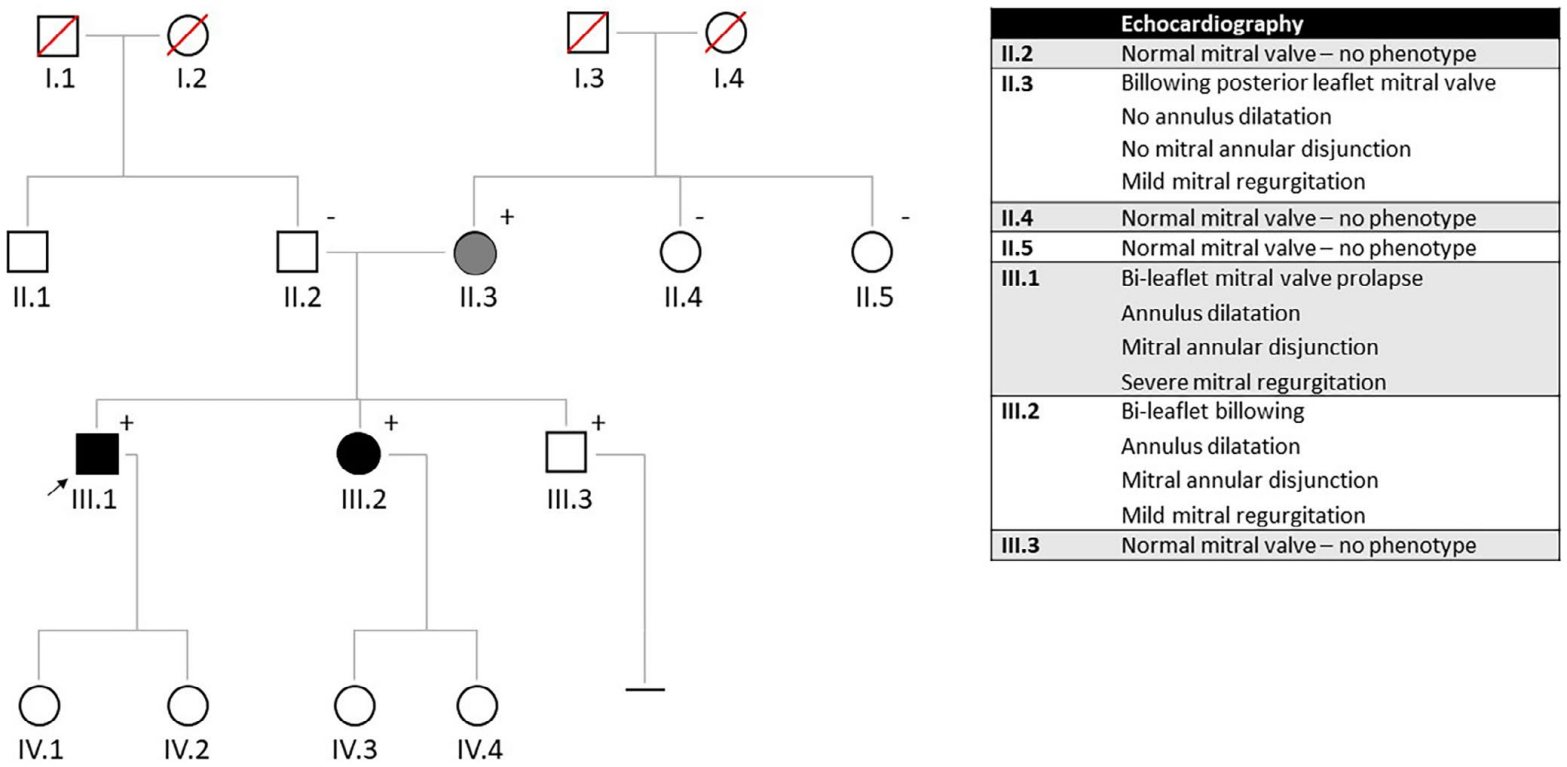
Family pedigree. Squares/circles indicate male/female family members, respectively; open symbols represent unaffected persons; symbols with a slash represent deceased persons; solid symbols represent affected persons (black represents a severe phenotype and gray a mild phenotype). The proband (III.1) is indicated by the arrow. PDLIM7 variant carriers are indicated by a plus sign. The numbers below the subject symbol denote the identification of the family members used in the text.

The family described in this report consisted of three members with MVP or billowing of the MV (mild form of the phenotype) (Figure 1). The index patient (III.1) was referred to the Department of Clinical Genetics, and his first‐degree family members were advised to undergo cardiac screening. Ultrasonography of the heart revealed a phenotype in his sister (III.2) and his mother (II.3), but not in his brother (III.3) or father (II.2). Since this suggested that the genetic cause would come from the mother, the sisters of the mother (II.4 and II.5) were also screened, and the brother of the father (II.1) was left out of the screening.

### 2.1 Patient 1

**FIGURE 2:**
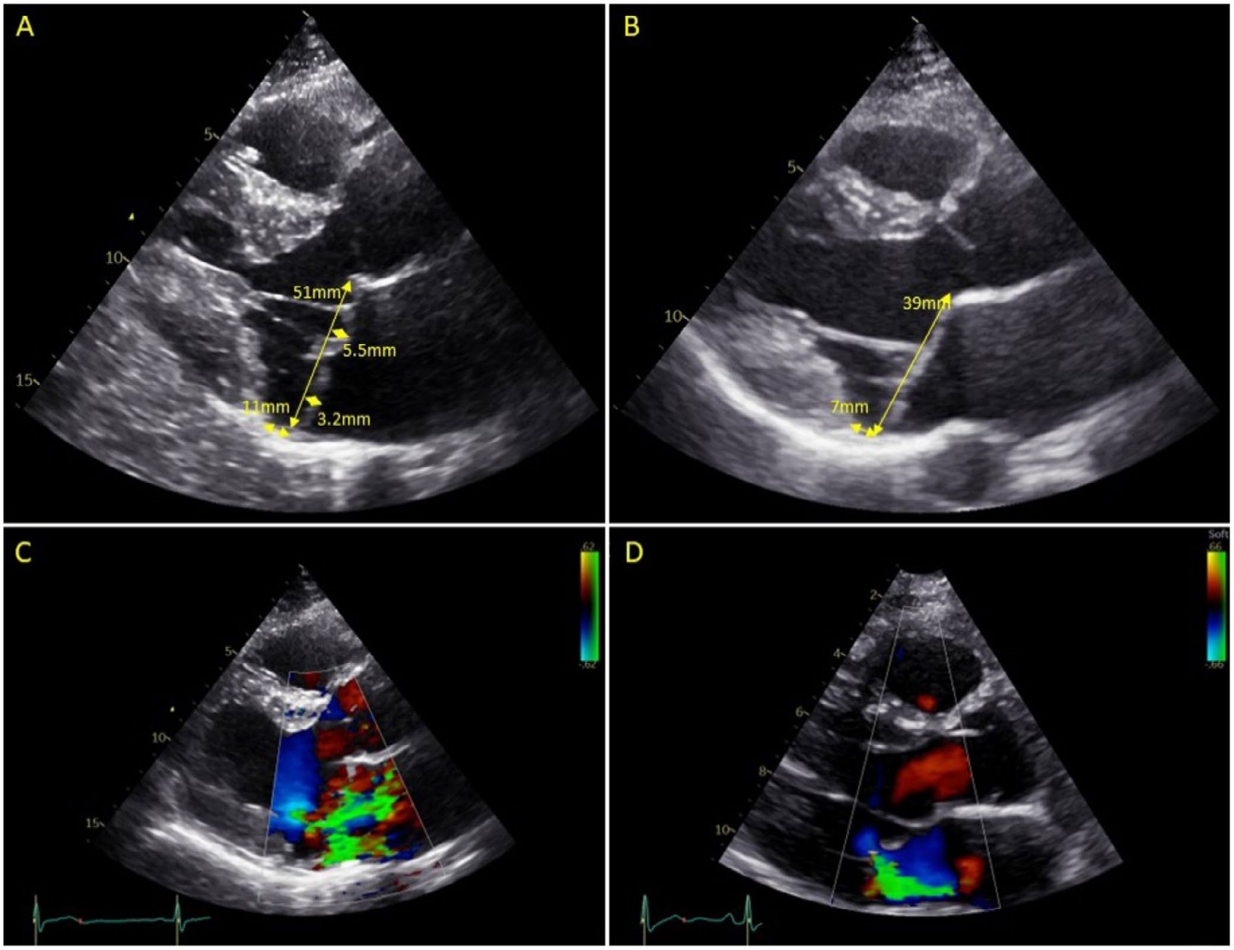
Echocardiograms of the family. (A) Echocardiogram of the proband (III.1) with Barlow's disease showing bi‐leaflet prolapse (> 2 mm), annular dilatation (51 mm) and mitral annular disjunction (11 mm) and (B) echocardiogram of the sister (III.2) with bi‐leaflet billowing, annular dilatation (39 mm) and mitral annular disjunction (7 mm). (C) Color echocardiogram of the proband showing severe mitral regurgitation and (D) color echocardiogram of the sister showing mild mitral regurgitation.

A 38‐year‐old male (III.1) was referred to our Department of Clinical Genetics after cardiac surgery due to MVP with severe mitral regurgitation. He was already known with MVP for 17 years, and he was seen on a regular basis by his cardiologist. Physical examination revealed no signs of connective tissue disorders. Over time, his mitral regurgitation progressed and became severe, and he had been referred to our Cardiology Department for evaluation prior to cardiac surgery. The echocardiogram before surgery revealed characteristics of Barlow's disease, including MV bi‐leaflet prolapse due to excessive tissue, severe MV annular dilatation, and mitral annular disjunction (defined as an atrial implantation of the MV posterior leaflet), together with severe mitral regurgitation and left atrial dilatation (Figure 2A,C). The other cardiac valves (aortic, tricuspid, and pulmonary valve) showed no abnormalities. He was therefore referred for surgical MV repair, which was successful and without complications. First‐degree family members were advised to undergo an echocardiogram to screen for MVP.

### 2.2 Patient 2

The younger sister of the proband (III.2) was 34 years old when she underwent an echocardiogram. She had no medical history and no symptoms. Based on the echocardiogram, she was also diagnosed with Barlow's disease (bi‐leaflet prolapse, MV annular dilatation and mitral annular disjunction) but with mild mitral regurgitation (Figure 2B,D). The aortic, tricuspid, and pulmonary valves showed no abnormalities. Physical examination revealed no signs of connective tissue disorders. She has now been seen on a regular basis by her cardiologist for follow‐up.

### 2.3 Patient 3

The mother of the proband (II.3) was screened at the age of 58 years, and her medical history revealed only hypertension. The echocardiogram showed billowing of the MV posterior leaflet with trivial mitral regurgitation. The MV annulus was not dilated and showed no signs of mitral annular disjunction. The other cardiac valves were normal.

### 2.4 Other Family Members

The brother of the proband (III.3), the father of the proband (II.2) and the two sisters of the mother (II.4 and II.5) all had a normal echocardiogram, and no cardiac valve abnormalities were seen. The underaged children of the proband (III.1) and his sister (III.2) were not yet screened.

## 3 Methods

During the genetic counseling, informed consent was obtained from all family members for molecular analysis and publication of the data. In this family, the genetic screening was first performed in the two individuals with the clearest phenotype, the index (III.1) and his younger sister (III.2). Both were diagnosed with the most severe form of degenerative MV disease, namely Barlow's disease (bi‐leaflet MVP due to excessive tissue, severe MV annular dilation and mitral annular disjunction).

DNA was extracted from peripheral blood according to standard protocol. Variant filtering and interpretation were performed on the exome data using the Leiden Open Variation Database (LOVDplus V.3.0. Build 28c, Leiden, the Netherlands). Variants with a quality score < 100 and variants with an allele frequency > 5% in the LOVD (in house) database were removed. The variants that remained after filtering were analyzed and checked for occurrence in the population databases Genome Aggregation database (GnomAD v4.1.0). Moreover, to establish whether a variant was reported before, the Human Gene Mutation Database (HGMD Professional) and the ClinVar database were consulted. The functional annotation algorithms SIFT, PolyPhen‐2 HumVar, and Align GVGD were used to predict the deleterious effects of missense variants, and to determine the effect of a variant on splicing, the use of splice prediction programs integrated in Alamut (Interactive Biosoftware Version 1.10, Rouen, France) was used. The PhyloP score was used to evaluate conservation between amino acid substitutions among species. The Classification of the variants was performed according to the American College of Medical Genetics and Genomics guidelines. Variants were reported according to guidelines available from the Human Genome Variation Society. Literature studies and data in Online Mendelian Inheritance in Man were used to look for relations between the genes and heart valve development of disease.

By comparing the whole exome of the index and his affected sister, 981 shared variants were identified. Variants with a low PhyloP value (< 2.0, indicating weak evolutionary conservation) and a high frequency in population databases (> 0.02%) and our in‐house database (≥ 20 hits) were excluded. This resulted in 27 remaining shared variants, which were further analyzed. Of these 27 variants, only one gene was known to be associated with cardiac development and valve abnormalities (specifically MV abnormalities), namely the *PDLIM7* gene.

## 4 Conclusion and Results

The variant in *PDLIM7* was a missense variant (NM_005451.5: c.716C>T p.(Thr239Met)) (AMCG classification: variant of unknown significance—PM2 and PP3). A literature search on *PDLIM7* revealed that the gene was involved in cardiac valve formation. The frequency of the variant was very rare in the population database GnomAD v4.1.0 (0.002%) and the variant was absent in our in‐house database. The variant involved a moderately conserved nucleotide (PhyloP 5.48) and amino acid (considering 12 species) and the functional annotation algorithms Polyphen2 and SIFT predicted a probably damaging or deleterious effect of the variant. By testing the other family members (the first‐degree family members of the index and the first‐degree family members from the mother) specifically on this variant, co‐segregation was shown, even though one carrier (III.3) showed no phenotype at the age of 29. However, incomplete penetrance and variable age of onset are common in genetic cardiac disorders.

## 5 Discussion

We report a family with MVP, so far presenting no ventricular arrhythmias, and a missense variant in *PDLIM7* co‐segregating with the phenotype and predicted to be likely pathogenic by several functional annotation algorithms; therefore, we suggest *PDLMI7* as a possible novel candidate gene for familial MVP.

The function of PDLIM7 was previously investigated in zebrafish and mice. In zebrafish, PDLIM7 transcripts were detected in the developing heart. These zebrafish models also revealed that PDLIM7 is able to regulate the subcellular localization and transcriptional activity of TBX5 [14]. TBX5 travels back and forth between the nucleus and the cytoplasm. In the cytoplasm, TBX5 can bind to PDLIM7 along the actin filaments. The binding of TBX5 to PDLIM7 prevents TBX5 from returning to the nucleus and activating target genes [15]. *TBX5* is a gene known to be involved in cardiovascular development in humans, and *TBX5* mutations result in cardiac malformations as seen in Holt‐Oram syndrome [17]. Specifically, Holt‐Oram syndrome is caused by missense mutations in the *TBX5* gene, resulting mostly in a loss of function of the TBX5 protein, leading to upper limb and cardiac malformations, including MV abnormalities such as MVP [18, 19, 20]. Knockdown of PDLIM7 in zebrafish resulted in developmental heart malformations due to failure of looping of the developing heart. The failure of looping of the developing heart could be due to TBX5 misregulation in the actin filaments because of loss of PDLIM7 function. More specifically, knockdown of PDLIM7 in zebrafish embryos resulted in the absence of valve tissue despite normal formation of the other cell layers (the myocardial and endocardial cell layer). On the contrary, lack of TBX5 function while PDLIM7 function was intact led to increased valve leaflet tissue compared to wild‐type zebrafish hearts [15]. Theoretically, a mutation in PDLIM7 resulting in inhibition of TBX5 could therefore lead to valve abnormalities, including MVP.

In mice, the presence of PDLIM7 transcripts was confirmed in different regions of the heart: the atria, trabeculated regions of the ventricles, and the atrial and interventricular septa. Moreover, the PDLIM7 protein was found in the developing atrioventricular and outflow tract cushions of the heart. PDLIM7 knock‐out mice showed normal early cardiac valve development. However, echocardiography on 3‐month‐old PDLIM7 knock‐out mice showed increased mitral and tricuspid valve annulus diameters compared to wild‐type mice. Histological analysis of the mitral and tricuspid valves of the PDLIM7 knock‐out mice showed an elongation of the mitral and tricuspid valve leaflets, suggesting abnormal remodeling of the mitral and tricuspid valves in the absence of PDLIM7 at a later stage of development [16]. A possible explanation could be that PDLIM7 has a yet unknown function in epicardial‐derived cells that supports valve remodeling [21].

Both an increased valve leaflet tissue, as seen in zebrafish with inhibition of TBX5, as well as MV annular dilatation and elongation of the MV leaflets, as seen in PDLIM7 knock‐out mice, are typical features seen in patients with MVP, especially Barlow's disease. Therefore, these results of PDLIM7 in zebrafish and mice, in addition to evidence coming from *TBX5* mutation studies, indicate that there could also be a role for PDLIM7 in valvular abnormalities in humans. The current family with familial MVP and a co‐segregating missense variant in the *PDLIM7* gene is a first indication of such a possibility, even though further research and functional studies will be necessary to confirm the causal relationship. Identifying novel candidate genes such as *PDLIM7* could have broader implications, including potential impacts on diagnosis, treatment, and genetic counseling.

## Author Contributions

**Aniek L. van Wijngaarden:** conceptualization, methodology, project administration, writing – original draft. **Tamara T. Koopmann:** writing – review and editing. **Claudia A. L. Ruivenkamp:** writing – review and editing. **Hoi W. Wu:** writing – review and editing. **Nina Ajmone Marsan:** conceptualization, writing – review and editing. **Daniela Q. C. M. Barge‐Schaapveld:** conceptualization, writing – review and editing.

## Ethics Statement

The authors have nothing to report.

## Consent

We obtained written informed consent from all three patients to publish this case series in accordance with the journal's patient consent policy.

## Conflicts of Interest

The authors declare no conflicts of interest. The Department of Cardiology of Leiden University Medical Center received unrestricted research grants from GE Healthcare, Medtronic, Boston Scientific, Biotronik, and Edwards Lifesciences. Nina Ajmone Marsan received speaker fees from GE Healthcare and Abbott Vascular.

## Data Availability

The data that support the findings of this study are available on request from the corresponding author. The data are not publicly available due to privacy or ethical restrictions.
